# Towards Reliable Healthcare Imaging: A Multifaceted Approach in Class Imbalance Handling for Medical Image Segmentation

**DOI:** 10.1007/s12539-025-00726-2

**Published:** 2025-07-07

**Authors:** Lijuan Cui, Mingquan Xu, Chao Liu, Tianyu Liu, Xiaoting Yan, Yan Zhang, Xiaofeng Yang

**Affiliations:** 1 College of Computer Science and Technology (College of Data Science), Taiyuan University of Technology, Taiyuan, 030024 China; 2 First Clinical Medical College, Shanxi Medical University, Taiyuan, 030001 China; 3 Biomedical Engineering Research Center, First Hospital of Shanxi Medical University, Taiyuan, 030001 China; 4 School of Computer Science, Northwestern Polytechnical University, Xi’an, 710072 China; 5 Department of Urology, First Hospital of Shanxi Medical University, Taiyuan, 030001 China; 6 School of Opto-electronic Engineering, Xi’an Technological University, Xi’an, 710021 China

**Keywords:** Class imbalance, Deep neural network, Medical image, Segmentation

## Abstract

Class imbalance is a dominant challenge in medical image segmentation when dealing with MRI images from highly imbalanced datasets. This study introduces a comprehensive, multifaceted approach to enhance the accuracy and reliability of segmentation models under such conditions. Our model integrates advanced data augmentation, innovative algorithmic adjustments, and novel architectural features to address class label distribution effectively. To ensure the multiple aspects of training process, we have customized the data augmentation technique for medical imaging with multi-dimensional angles. The multi-dimensional augmentation technique helps to reduce the bias towards majority classes. We have implemented novel attention mechanisms, i.e., Enhanced Attention Module (EAM) and spatial attention. These attention mechanisms enhance the focus of the model on the most relevant features. Further, our architecture incorporates a dual decoder system and Pooling Integration Layer (PIL) to capture accurate foreground and background details. We also introduce a hybrid loss function, which is designed to handle the class imbalance by guiding the training process. For experimental purposes, we have used multiple datasets such as Digital Database Thyroid Image (DDTI), Breast Ultrasound Images Dataset (BUSI) and LiTS MICCAI 2017 to demonstrate the prowess of the proposed network using key evaluation metrics, i.e., IoU, Dice coefficient, precision, and recall.

**Graphical Abstract:**

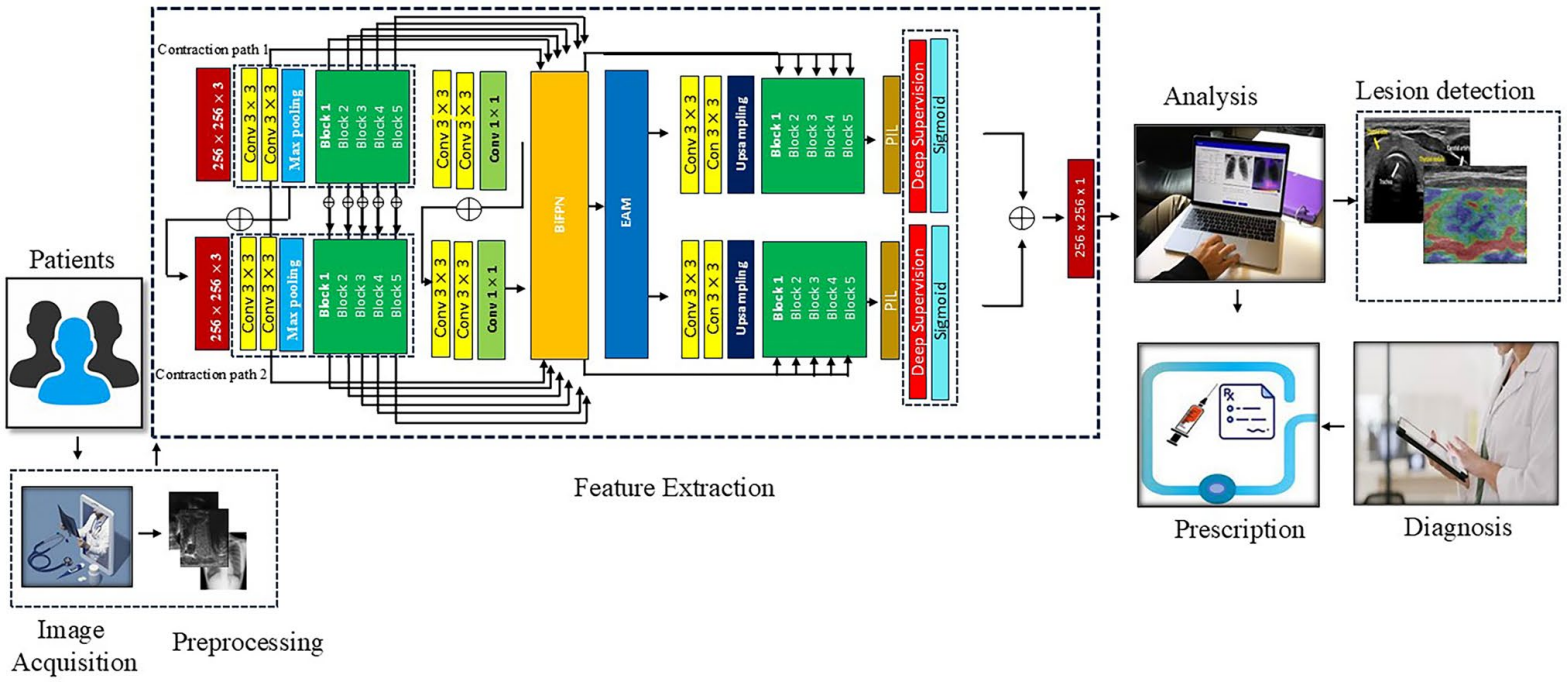

## Introduction

Medical image segmentation plays a critical role in histopathology and clinical assessment, enabling the division of digital images into focal areas such as tumors, organs, or anatomical structures. The development of traditional radiography to advanced imaging techniques like MRI and CT scans has significantly improved image resolution and quality. However, these advancements have also introduced challenges, particularly in ensuring accurate and reliable segmentation of Region of Interest (ROI). One of the most persistent challenges in this area is class imbalance, where certain classes (e.g., specific types of tissue or pathological features) have rare representation in datasets. This imbalance leads to biases in segmentation models, causing them to overlook or misclassify minority classes, which are often clinically significant. For instance, in oncology, lesions are typically smaller than surrounding tissues, making them prone to being underrepresented in datasets [1, 2].

Class imbalance poses a significant challenge in developing robust segmentation algorithms. Traditional deep learning models, such as Convolutional Neural Networks (CNNs) [3, 4], excel in image recognition and segmentation tasks when trained on extensive and diverse datasets. However, if the training data lacks sufficient relevance or diversity, the model's performance may become suboptimal. These models tend to favor majority classes, leading to poor performance in detecting minority classes [5–7]. This issue is particularly critical in medical imaging, where accurate segmentation of underrepresented classes (e.g., small lesions or rare tissues) is essential for accurate diagnosis and treatment planning.

Recent strategies for handling class imbalance can be classified into three categories: data-centric methods, algorithm-driven techniques, and hybrid approaches. Data-centric or data-level approaches, such as undersampling majority classes or oversampling minority classes, strive to balance class distribution but often face challenges like limited data availability and potential bias [1, 8–10]. Algorithm-driven approaches, such as cost-sensitive learning [9, 11–13] and cascade training [6, 14–16], modify the learning process to reduce bias toward majority classes. Hybrid methods combine data-level and algorithm-driven strategies to overcome the limitations of individual approaches, leading to improved accuracy and robustness in segmentation models [17–20]. Several studies have proposed methodological strategies to address this issue, including two-stage approaches[21–23] and semi-automatic methods [24]. In two-stage methods, ROIs are first detected to narrow down the areas surrounding lesions, followed by segmentation in the subsequent stage. Conversely, semi-automated methods rely on human input, where individuals manually draw a bounding box around the lesion to establish an initial estimate of its position, making the subsequent segmentation task easier. Although these techniques provide ways to address class imbalance, they have drawbacks: semi-automated methods depend on human effort, while two-phase strategies risk losing surrounding contextual details, potentially reducing the accuracy of the final outcome.

In this study, we introduce a hybrid approach to tackle class imbalance in medical image segmentation. Our proposed method combines strategies at the data, algorithm, and methodological levels to mitigate the class imbalance challenges encountered by deep learning models.

Our proposed model, Dual Decoder UNet (2D-UNet), integrates both data-level and algorithm-level strategies to effectively handle imbalanced datasets**.** At the data level, we introduce a multi-dimensional data augmentation technique specifically designed for medical imaging, which enhances the representation of minority classes and reduces bias toward majority classes**.** At the algorithm-driven level, we propose a novel hybrid loss function that assigns greater weight to minority classes, ensuring that the model pays adequate attention to underrepresented regions during training. Additionally, we incorporated Enhanced Attention Module (EAM) and spatial attention mechanism to focus the model on the most relevant features, particularly those of minority classes. Unlike traditional two-stage approaches that rely on an initial detection phase to localize lesions and reduce contextual noise, our dual decoder system achieves this within a single framework: one decoder focuses on lesion regions (foreground), while the other captures contextual background details, and their outputs are combined via the Pooling Integration Layer (PIL) to produce a refined segmentation, enhancing performance on imbalanced medical datasets.

The architecture also includes a dual decoder system to capture accurate foreground and background details, ensuring balanced feature extraction.

The key contributions of this research include:

- **Novel Attention Mechanism**: We developed an EAM and spatial attention mechanisms to focus on ROIs of various sizes and shapes, particularly addressing underrepresented classes.
- **Incorporation of BiFPN in 2D-UNet**: We integrate a Bi-directional Feature Pyramid Network (BiFPN) into the 2D U-Net architecture to enhance multi-scale feature extraction, ensuring balanced representation of all classes.
- **Dual Decoders for Segmentation**: We implement two distinct decoders for simultaneous foreground and background segmentation, improving the model's ability to handle class imbalance.
- **Proposed Hybrid Loss Function**: We designed a hybrid loss function that directly addresses class imbalance by assigning greater weight to minority classes during training.
- **Pooling Integration Layer (PIL)**: The PIL ensures efficient utilization of both global and local features, minimizing feature loss and improving segmentation accuracy for minority classes.

The structure of this article is as follows: Sect. 2 reviews related studies, Sect. 3 outlines the proposed methodology, Sect. 4 analyzes the results and discusses the effectiveness of the proposed approach, and Sect. 5 concludes the work.

## Related Work

A multifaceted CNN model for medical image segmentation refers to a deep-learning architecture designed to segment medical images [25, 26] into different ROIs. Over the past few years, numerous research efforts have addressed the issue of class and data imbalances in classification and segmentation tasks using deep learning models. This section presents a review of the significant research contributions in this domain.

In [27], the authors employed the Class Attention to Regions of the Lesion (CARE) framework to address class and data imbalances by incorporating specialized attention during the CNN model's training process. This attention mechanism is only active during training and does not alter the original network's structure. As a result, it can be seamlessly integrated into any existing CNN architecture.

To address class imbalance in histopathological datasets, [28, 29] employed a spatially aware CNN namely SPNet. This model's cost function focuses on specific spatial regions, leading to a 1.9% improvement in F1 classification for rare class types in the CoNSeP dataset. In [30, 31], researchers utilized deep learning models to diagnose osteoarthritis from medical images. They investigated various techniques to address the challenge of class imbalance in the dataset in order to improve models’ performance. Research conducted by [29, 32, 33] investigated the challenge of imbalance in COVID-19 images and cellular nuclei datasets derived from histopathological images.

In [29], the authors introduced a nuclei segmentation model designed to address imbalances in the data. They proposed an optimized lightweight U-Net based segmentation network for this purpose and evaluated the model's performance using metrics such as the Intersection of Union (IoU) and the Aggregated Jaccard Index (AJI). In [32], researchers aimed to mitigate loss and class imbalances using a federated learning approach. They introduced a context aggregator federated learning framework, specifically evaluated on COVID-19 imaging datasets. The results indicated superior performance compared to conventional federated averaging algorithms. Furthermore, [34] introduced a hybrid sampling technique to address data imbalance in Raman spectroscopy. This approach integrates Raman-Gaussian distributed oversampling with random undersampling. It was implemented on datasets comprising malignant tumors, class B infectious diseases, and autoimmune disorders.

Several studies [27, 35, 36] have explored the adoption of a multi-task delineation strategy to streamline intricate multi-class segmentation tasks. This approach decomposes the problem into smaller, more manageable tasks, where each training phase concentrates on segmenting a specific region. By doing so, it helps achieve a more balanced label distribution compared to simultaneously segmenting multiple classes. In [37, 38], class imbalance was mitigated, using ensemble learning techniques and semi-supervised federated learning, respectively. The ensemble approach integrates multiple classifiers, which can be either identical or diverse, to enhance generalization and address data imbalance. Unlike simpler models, employing cost-sensitive adjustments or resampling strategies in complex architectures may inadvertently result in overfitting, particularly when dealing with limited data from minority classes.

In [39, 40], the impact of training deep neural networks on imbalanced binary classification tasks was examined. The findings revealed that errors from the majority class largely affect gradient-based weight updates during training. To alleviate bias in CNN models, techniques such as cost-sensitive adjustments and instance resampling within mini-batches are commonly employed [39]. The synthetic resampling methods, especially those leveraging SMOTE [41], are often preferred due to their simplicity and classifier-independent nature as pre-processing steps. The data pair Generative Adversarial Network (GAN) was developed by [42] for synthesizing a wide range of medical images along with corresponding delineation labels from random latent vectors, addressing the challenge of limited pixel-wise annotations. Experimental results on vestibular schwannoma, kidney tumor, and skin cancer datasets demonstrate higher generative quality and improved semantic segmentation outcome juxtaposed with state-of-the-art GAN-based approaches. In [43], the author handled missing values and imbalance classes using the optimal amalgamation of imbalance treatment, imputation, amputation, and categorization based on classification performance. Meanwhile, [5] explored the schemes to address the class imbalance in auto-contouring oropharyngeal tumors in radiotherapy using deep learning methods, comparing multiple loss functions [44] and employing a two-phase method. During the analysis, no significant differences were found among the loss functions. The two-phase method significantly bested 3D U-Net in terms of segmentation accuracy, with a median Dice coefficient of 0.64, a Hausdorff distance of 8.7 mm, and a mean surface distance of 2.1 mm. Another work carried out by the CLDL (Curriculum Label Distribution Learning) Network was designed by [45] to tackle the label distribution imbalance issue in semantic delineation tasks. Employing R-LDL (Region Label Distribution Learning), a task-oriented curriculum learning strategy (TCL), and a prior perceiving module (PPM) improved the segmentation accuracy. Evaluation of the suggested model on MM-WHS2017 and BRATS2018 datasets showed significant enhancements over extant methods across numerous delineation metrics. A confidence interval-based balanced random learning scheme [46] was introduced to enhance the performance of CNN-based Landsat image isolation by mitigating the impact of noisy and imbalanced labels. Another study performed by [47] to present an active learning model tailored for fault detection in industrial images, addressing challenges such as imbalanced data and high labeling costs.

Class imbalance is often tackled by altering training strategies or input data sampling techniques, with less focus on refining the loss function. However, existing studies indicate that widely used approaches, such as upsampling underrepresented classes, tend to elevate false positive rates. Some methods incorporate attention mechanisms to enhance focus on minority classes. Additionally, training complex autoencoder-decoder networks requires substantial computational resources. Despite their effectiveness, these techniques lack selective induction, causing the network to prioritize globally prominent features while disregarding secondary yet equally significant features with slightly lower weight values. Likewise, the self-attention mechanism poses a challenge due to its high computational cost. In medical imaging, accurately segmenting ROIs is critical to minimizing missed diagnoses and improving diagnostic precision. Therefore, both dominant and subtle features play an equally important role in the segmentation process. Beyond architectural refinement, optimizing objective functions is also vital, as they directly impact the model's learning capabilities. To overcome these challenges, we propose a comprehensive approach that integrates both data-driven and algorithm-driven techniques. This approach is designed to address the shortcomings of existing methods, offering a more advanced and effective way to enhance relevant features while suppressing less significant ones in medical image analysis.

## Material and Method

### Datasets

To evaluate the proposed 2D-UNet, three different datasets were selected: Breast Ultrasound Images Dataset (BUSI) [48]. Digital Database for Thyroid Images (DDTI) [49], and LiTS MICCAI 2017 [50]. A detailed description of each dataset is given below.

#### DDTI

**Fig. 1:**
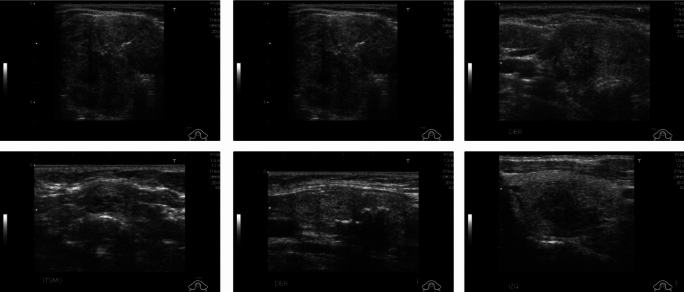
Sample images from the image data repository of DDTI

The DDTI dataset is an image data repository of ultrasonographic images annotated to find early thyroid cancer detection and diagnosis. The DDTI dataset contains B-mode ultrasound imaging, which is very important for researchers focusing on thyroid abnormalities. It comprises a total of 298 ultrasound images, with a gender distribution of 270 female and 29 male subjects. Each image in the dataset is precisely annotated with detailed diagnostic descriptions, enhancing its utility for educational and research purposes. This dataset not only supports the development of diagnostic algorithms but also provides variety of imaging conditions and thyroid sizes which is crucial for robust algorithm training. Annotations include information about the presence of nodules, their size, and their classification as benign or malignant. This annotation makes the DDTI dataset a comprehensive resource for training and validating machine learning models in medical imaging. For segmentation tasks, the imbalance is between nodule pixels (minority class) and non-nodule/background pixels (majority class). Thyroid nodules are generally small relative to the entire ultrasound image. The nodule area in this dataset is approximately 5%–15% of the image area, with significant variation based on nodule size. This implies a pixel-level imbalance of roughly 1:6 to 1:20 (nodule vs. background). The DDTI dataset can be accessed at https://www.kaggle.com/datasets/dasmehdixtr/ddti-thyroid-ultrasound-images, where it is available for public use. Figure 1 shows the visual representation of DDTI dataset sample images.

#### LiTS MICCAI 2017

**Fig. 2:**
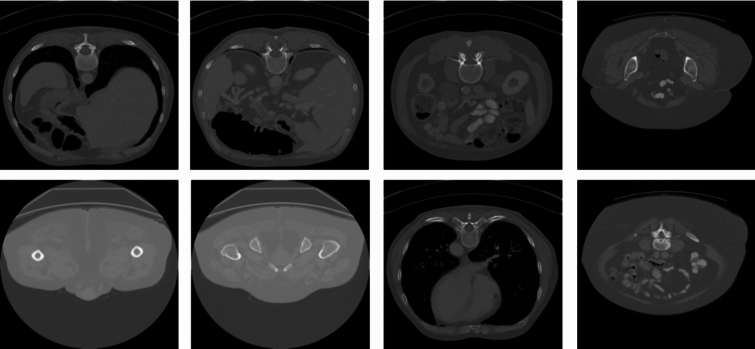
Sample images from the data repository of LiTS MICCAI 2017

The LiTS MICCAI 2017 dataset, or Liver Tumor Segmentation Benchmark, consists of 201 CT scans, primarily focused on the abdomen to aid the development and testing of CNN-based algorithms for liver tumor segmentation. This dataset, collected from various medical institutions, plays a crucial role in evaluating segmentation techniques by providing a varied array of liver conditions. Out of these, 194 scans reveal different types of liver lesions, enhancing the dataset's utility in addressing a broad spectrum of liver diseases. The LiTS dataset features cases of primary liver cancers such as hepatocellular carcinoma, cholangiocarcinoma, and metastatic liver diseases from colorectal, breast, and lung cancers, providing a rich basis for algorithm development and testing. In LiTS MICCAI 2017 tumor pixels constitute roughly 1%–5% of the total liver volume in most cases, with some volumes having even smaller tumor proportions (e.g., <1%) due to small lesions. An approximation for liver tumor datasets is 1:50 (2% tumor vs. 98% non-tumor) as a rough average, though small tumors can push this toward 1:100 or more. The graphical depiction of these images is presented in Fig. 2. The dataset is publicly available at https://www.kaggle.com/datasets/andrewmvd/lits-png/data.

#### BUSI

**Fig. 3:**
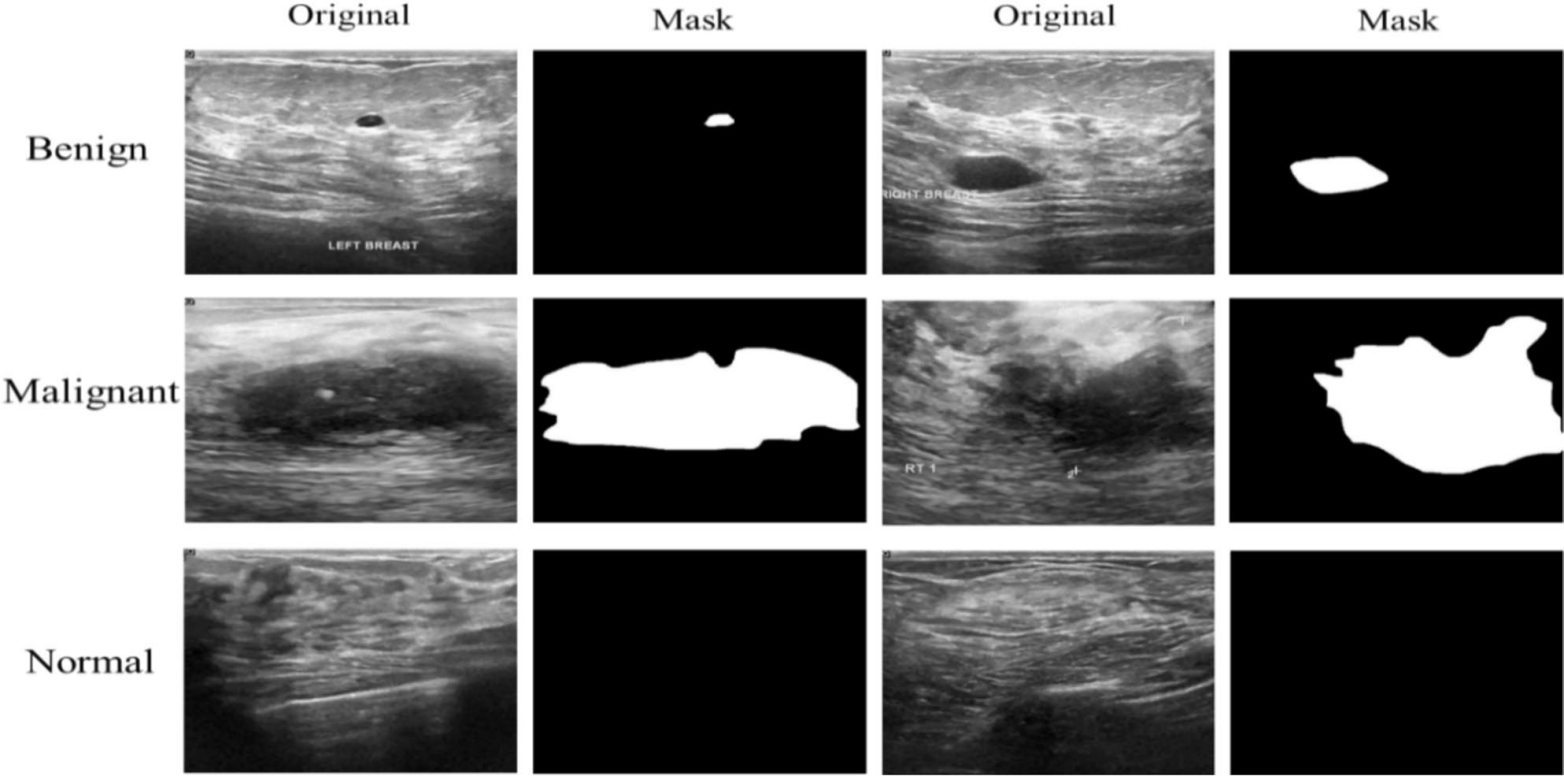
Benign, malignant, and normal sample images from the BUSI

The BUSI dataset is an important image repository for medical image segmentation research, specifically targeting breast ultrasound images along with their accurate ground truth segmentations. This dataset includes 780 breast ultrasound images sourced from female patients aged 25 to 75 years. Each image is provided in a 500 $\times$ 500-pixel PNG format, accompanied by precise ground truth data. The dataset includes 487 benign, 210 malignant, and 133 normal ultrasound images. This dataset is widely used for breast lesion segmentation and classification. Studies using BUSI report lesion areas roughly 5%–20% of the image, depending on lesion size. This shows a pixel-level imbalance of 1:4 to 1:20, with smaller lesions pushing toward the higher end as shown in Table 1. This dataset is accessible for public use and can be found on Kaggle at https://www.kaggle.com/datasets/aryashah2k/breacst-ultrasound-images-dataset. Representative images from the database can be viewed in Fig. 3.

**Table 1:** A statistical summary of imbalance ratios in medical imaging datasets

Dataset	Sample level imbalance	Pixel level imbalance
DDTI	2:1 (benign: malignant)	About 1:9 (10% nodule vs. 90% background)
LiTS MICCAI 2017	Not applicable (segmentation)	About 1:50 (2% tumor vs. 98% liver/background)
BUSI	2:1 (benign: malignant)	About 1:9 (10% lesion vs. 90% background)

### Pre-processing

The phase is critical to ensure that the input data is favorable to high-performance outcomes when subjected to the complex operations of a deep learning model like 2D-UNet. This section details the various pre-processing strategies employed to prepare the data for effective training and evaluation.

#### Data Acquisition and Normalization

The databases utilized, including LiTS MICCAI 2017, DDTI, and BUSI , consist primarily of high-resolution PNG images with varying dimensions and pixel densities. To standardize the input, we have performed resizing and normalization on the input images.

- **Resizing:** All images are adjusted to a consistent resolution of 256 $\times$ 256 pixels to maintain uniformity across datasets and reduce computational burden during training.
- **Normalization:** Values of pixels are scaled to [0,1] by dividing by 255. This normalization aids in stabilizing the learning process and improving the convergence speed of the neural network.

#### Data Augmentation

**Fig. 4:**
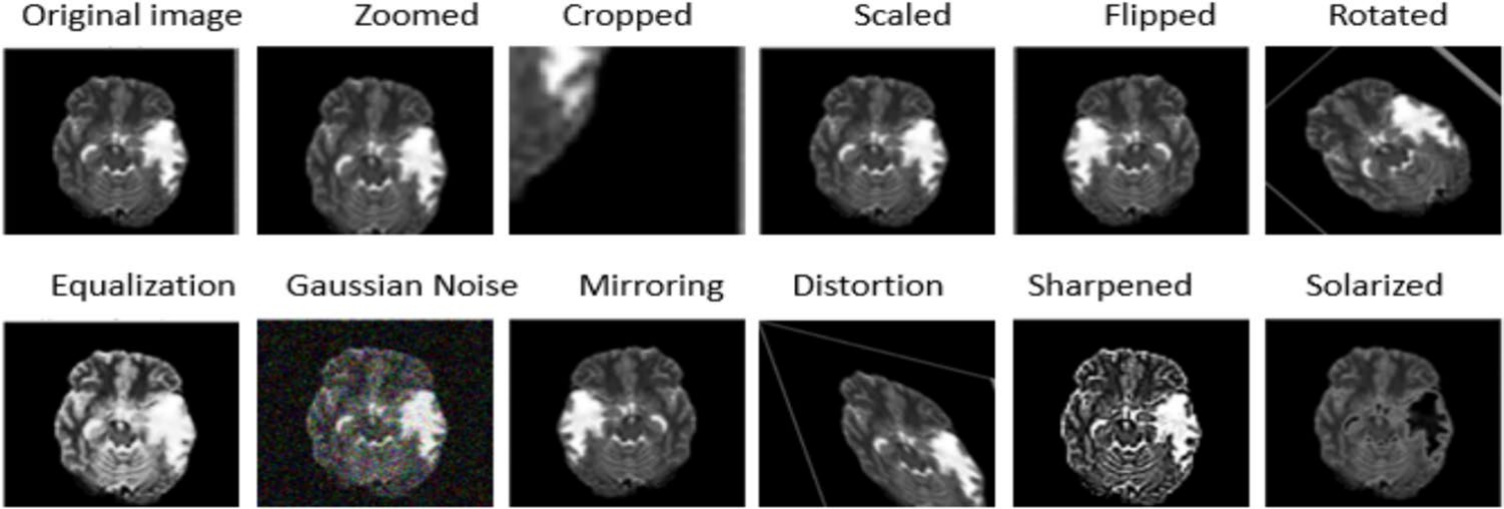
The outputs of various augmentation methods

Given the class imbalance and the variability in medical images due to different imaging conditions, data augmentation is implemented to artificially expand the training dataset. This includes:

- **Rotation**: Images are randomly rotated between −${10}^{\circ }$ and ${10}^{\circ }$ to simulate varying angles of scanning.
- **Translation**: Random translations are applied to simulate shifts that might occur during patient positioning.
- **Scaling**: Images are randomly scaled between 90% and 110% to account for size variations in the imaging field.
- **Flipping**: Vertical and horizontal flipping are employed to further boost the variability of the training data, representing different orientations of the anatomy. The graphical depiction of the augmented images is presented in Fig. 4.

#### Artifact Reduction

Medical images often contain artifacts that can obstruct the ability of models to extract relevant features effectively:

- Denoising: A Gaussian blur is applied to smooth out random noise in the images while preserving edge details critical for accurate segmentation.
- Contrast Enhancement: Histogram equalization is applied to enhance image contrast in order to make it easier for the model to differentiate between RoIs and the background.

#### Annotation Pre-processing

For segmentation tasks, corresponding ground truths are provided with each image. These annotations undergo the following adjustments to match the pre-processed input images:

- Binary Masks: For segmentation tasks, ground truth annotations are converted into binary masks where the pixels belonging to ROIs are marked as 1 (foreground) and all other pixels as 0 (background).
- Matching Transforms: All transformations applied to the input images are identically applied to the ground truth masks to maintain the alignment between the input data and labels.

### Method Overview of the Proposed Methodology

The proposed 2D-UNet consists of an encoder and dual decoders tailored to independently process foreground (lesion) and background segmentation, thereby reducing the bias typically seen in single-decoder architectures. This dual-decoder strategy ensures that the network learns distinct feature representations for both classes, preventing the overshadowing of minority-class features by dominant background regions. To counteract feature loss, which disproportionately affects smaller lesions, we integrate BiFPN layers to enhance multi-scale feature extraction. BiFPN improves the fusion of spatially detailed low-level features and semantically rich high-level features, ensuring that lesions—regardless of their size—are adequately represented.

**Fig. 5:**
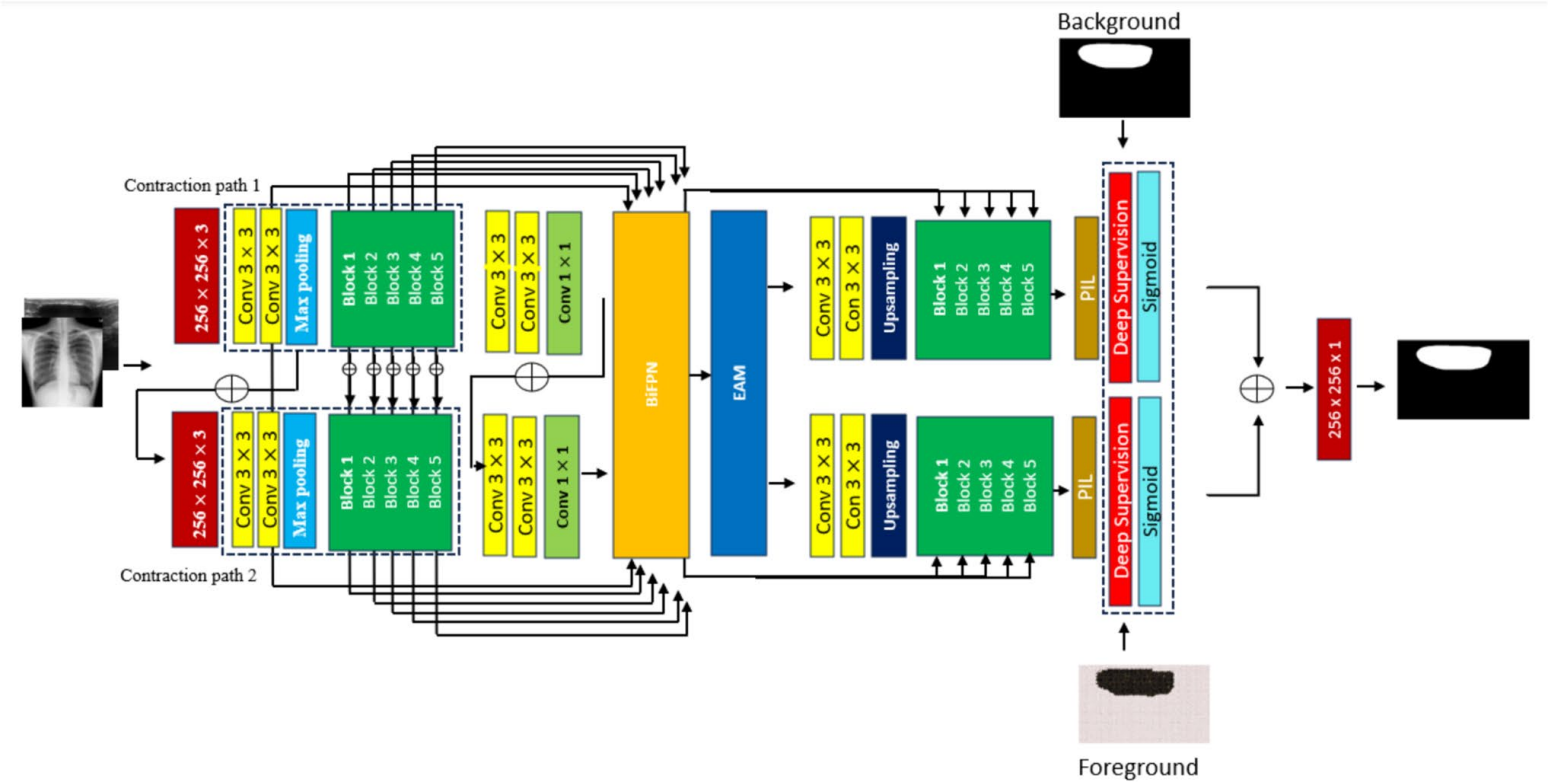
This diagram shows the internal structural details of the proposed 2D-UNet

Additionally, we introduce an EAM to amplify the focus on underrepresented lesion regions while suppressing irrelevant background information. This helps counteract the tendency of the model to ignore small or faint lesion areas, improving segmentation accuracy for minority-class regions. To further enhance the learning of minority classes, we incorporate a PIL, which efficiently merges low- and high-level feature representations. This integration ensures that lesion regions retain both semantic context and spatial accuracy, mitigating the resolution loss that often leads to segmentation errors in smaller classes. Finally, we employ a hybrid loss function that strategically assigns greater weight to minority-class pixels, counterbalancing the dataset’s inherent class imbalance. This ensures that underrepresented lesions contribute equally to the optimization process, improving segmentation performance for both majority and minority classes. A synoptic overview of the proposed model is illustrated in Fig. 5. A detailed description of each component of the model is be provided in the following sections.

#### Model Architecture

##### Encoder

The suggested network is a redefined classic UNet model. The redesigned encoder has an extra contraction path and is built with layers that are carefully constructed to enhance the network's ability to focus on accurately segmenting lesions in MRI images. This structure includes 3 $\times$ 3 and 1 $\times$ 1 convolutional layer, followed by batch normalization and the ReLU activation layer. To maintain the same size for both the input and output feature maps and ensure no loss of edge pixels, padded convolution with a stride of 1 is used.

Following the second convolution in each layer of the first contraction path, a maximum pooling layer (2 $\times$ 2) with a stride of 2 is applied. In the layers of the second contraction path, the feature maps from the corresponding layer in the previous contraction path are added to the feature maps from the current layer. This combined feature maps are then processed using a 3 $\times$ 3 convolution layers, followed by batch normalization and ReLU layers. The addition of feature maps from both paths enhances lesion segmentation. The BiFPN is inserted after the final feature extraction stage in the encoder, before passing features to EAM. It takes multi-scale feature maps from different layers of both contraction paths and refine them through weighted feature fusion before sending the enhanced features to the dual decoders. This improves the feature representation by ensuring effective cross-scale connections before the attention mechanisms are applied.

##### Dual Decoder

The dual branch decoders are an approach employed to enhance the performance of the model in segmented images specifically, in challenging areas where the difference between the background and ROIs is difficult to distinguish. The proposed dual decoders comprise two branches, one dedicated to background segmentation and the other for foreground segmentation. These decoders are designed to resemble the structure of the U-Net decoder, with each decoder consisting of successive convolutional layers using a 3 $\times$ 3 kernel size, upsampling to increase the resolution of the feature maps. This architecture enables the decoders to gradually increase the resolution of the feature maps while integrating semantic information from the enhanced feature map.

The last layer of the encoder generates a feature map with 1024 channels, each of which is a 32 $\times$ 32 representation of the input image. This layer has the largest receptive field in the encoder, which can capture information from a larger portion of the image, which is vital for understanding the overall context and structures within the image. To further refine multi-scale information, we integrate a BiFPN after the encoder. BiFPN efficiently fuses feature maps from multiple encoder layers, assigning learnable weights to balance the contribution of low-level spatial and high-level semantic features. This fusion ensures better feature propagation before passing the refined features to EAM. EAM then captures pixel-wise dependencies and enhances segmentation precision by emphasizing important spatial relationships.

We implement an EAM to capture the relationships between pixels within the feature maps. A detailed discussion on EAM is given in section d. This module computes an attention matrix that emphasizes the most significant spatial connections between various regions of the input image.

The output of EAM is generated by multiplying the original feature map with their transformed version. This process highlights the most important features and improves the model’s ability to focus on relevant parts of the image.

This map contains richer semantic information compared to ${\varvec{O}}_{M}\in {\mathbb{R}}^{1024\times 32\times 32}$, as it considers both the intrinsic features and their spatial relationships. To achieve two distinct segmentation results while retaining all semantic information, the network employs two separate decoders. These decoders mirror the structure of the U-Net decoder, each consisting of four consecutive convolutional layers with a kernel size of 3 $\times$ 3. The upsampling is applied that allows the decoders to progressively increase the resolution of the feature maps while incorporating the semantic information from the enhanced feature map.

The generated feature maps from the proposed model contain extracted information about the foreground and background from the input representation. We merge the feature maps from both decoders by applying the fusion operation, as shown in Eq. (1).

1 $$\begin{array}{c}{{\varvec{O}}}_{\text{Final}}=\varvec{1}-\frac{\text{sigmoid}\left(\left({{\varvec{M}}}_{\text{F}}\right)+\text{sigmoid}\left({{\varvec{M}}}_{\text{B}}\right)\right)}{2}\end{array}$$

where ${{\varvec{M}}}_{\text{B}}, {{\varvec{M}}}_{\text{F}} \in {\mathbb{R}}^{512\times 512}$ represent background and foreground features maps, respectively.

Training a model for various tasks, such as foreground and background segmentation, can sometimes be very challenging. To address this, we employ an integrated loss function to balance the training process for each decoder by assigning the most appropriate values to the hyperparameters. We find that assigning weights of $\alpha =0.6$, $\beta$ = 0.2 is optimal for both background and foreground losses. Moreover, we employ a final integration layer that combines the final segmentation layers from both decoders, as shown in Fig. 5. This final merger ensures that both decoders contribute to the overall loss function, resulting in the best performance for the final segmentation. The loss function for the final integration layer is shown in Eq. (2).

2 $$\begin{array}{c}l= \alpha {L}_\text{Backgound}+{\beta L}_\text{Foreground} \end{array}$$

##### PIL

Effectively distinguishing between different classes in segmentation requires ensuring intra-class similarity (consistency within the same class) and inter-class difference (clear separation between different classes) in feature maps. Low-level features retain spatial details but lack semantic accuracy, while high-level features provide semantic context but suffer from resolution loss. This imbalance leads to mismatches, disproportionately affecting smaller or less frequent classes, which are already underrepresented.

To counteract this, we refine high-level features using low-level guidance before fusion. Instead of directly merging these features where convolution layers may struggle to correct boundaries due to their uniform operations, we employ average pooling to smooth high-level features, reducing abrupt transitions. This process reinforces the representation of minority classes, preventing them from being overshadowed by dominant classes. By refining and improving feature integration, our method enhances segmentation accuracy, particularly for underrepresented classes, thereby alleviating class imbalance effects.

We have introduced a PIL to merge the abstract, high-level information and detailed, low-level information from input representation. PIL improves segmentation quality by systematically balancing spatial and semantic information. Unlike conventional approaches that either prioritize context or resolution, PIL harmonizes both aspects through a structured fusion mechanism, leading to enhanced boundary precision, better class distinction, and improved segmentation accuracy, especially for minority classes.

Prior to this integration, high level features are passed through the smoothing layer (i.e. 3 $\times$ 3 average pooling layer) that helps to remove any rough edges from high-level features. After smoothing, high-level features are combined with low-level details. The integrated feature set is then passed through 1 $\times$ 1 convolution, normalization and ReLU layers to improve feature maps that balances the need for differences between classes (e.g., distinguishing between different objects) and similarities within the same class (e.g., recognizing different parts of the same object).

##### EAM

Traditional deep learning-based medical image segmentation models classify each pixel independently into one of the possible *K *classes or categories, relying on per-pixel cross-entropy loss. However, the pixel-wise approach may overlook global contextual information, making it less effective for segmenting complex anatomical structures in MRI images.

**Fig. 6:**
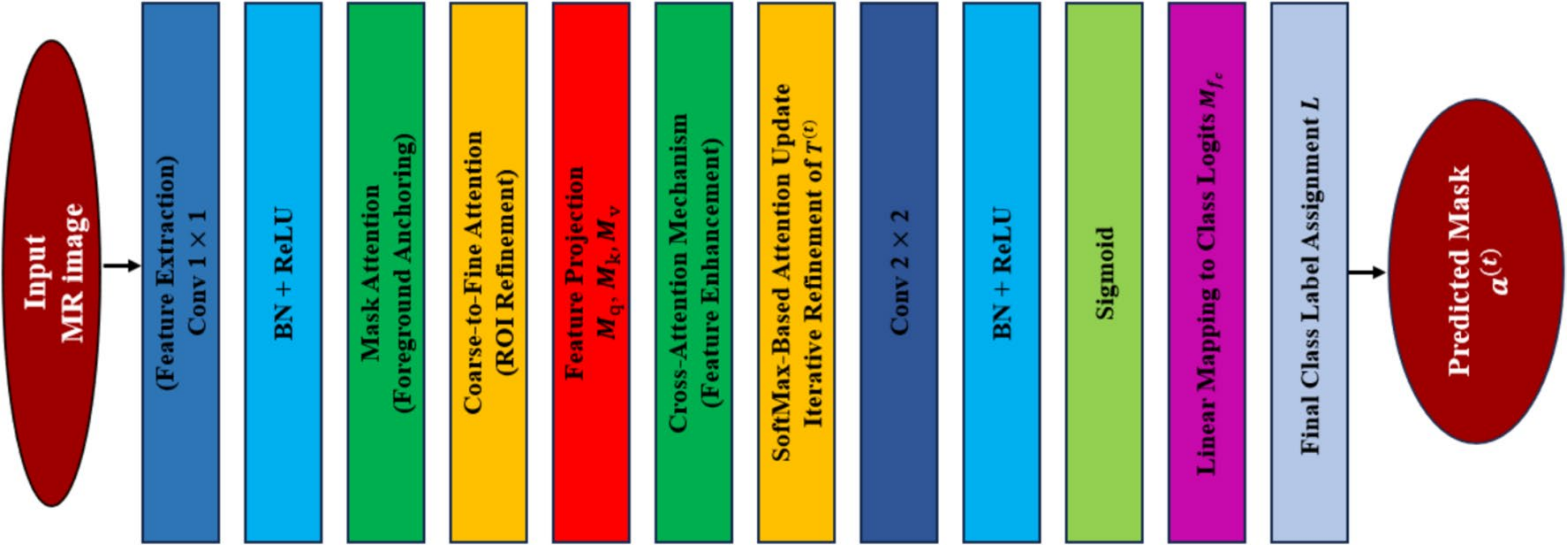
The synoptic overview of EAM

To improve the image delineation process, we introduce a novel attention module, namely EAM, specifically designed to reduce the negative impact of irrelevant background areas and mitigate model bias towards majority classes, ensuring better segmentation of underrepresented regions. EAM as shown in Fig. 6 epitomizes an improvement upon existing attention mechanisms by optimizing its limitations in traditional self-attention and coarse-to-fine segmentation methods.

The coarse-to-fine attention mechanism plays a crucial role in medical image segmentation, especially for identifying small ROIs that are difficult to delineate in MRI images. Unlike conventional attention mechanisms, EAM explicitly leverages an initial coarse mask prediction to guide attention in subsequent refinement stages. This helps the model focus on relevant anatomical structures while suppressing background noise.

The coarse prediction mask is first generated as an initial estimate of the segmented region and is later refined using attention-based mechanisms. This process progressively improves segmentation quality through iterative refinement, ensuring precise boundary detection. Mathematically, the initial segmentation prediction is computed as shown in Eq. (3).

3 $$\begin{array}{c}{{\varvec{T}}}^{(0)}=S({{\varvec{T}}}_\text{o}\times {{\varvec{Q}}}^\text{T}), \\ {{\varvec{T}}}_\text{o}\in {\mathbb{R}}^{ A \times D}, \varvec{Q}\in {\mathbb{R}}^{ A\times C\times H\times W\times D}\end{array}$$

where ${{\varvec{T}}}_\text{o}$ represents the initial set of segmentation candidates, while $\varvec{Q}$ denotes the embedding of the last feature set from the encoder. $A$ indicates the number of candidate areas in the input image, $C$ refers to the number of channels, $W$ represents the width, and $H$ denotes the height of the input image. $S$ represents a sigmoid function.

To progressively refine the coarse segmentation mask, we integrate a mask attention layer that anchors cross-attention within the foreground regions based on the coarse predictions for each class. By filtering out background noise, the model enhances its focus on ROIs, improving segmentation precision. At each iteration $t$, the segmentation refinement process is formulated as

4 $$\begin{array}{c}{{\varvec{T}}}^{(t+1)}={{\varvec{T}}}^{(t)}+\text{softmax }\left(\left({{\varvec{T}}}^{(t)}{{\varvec{M}}}_{q}\right){\left({\varvec{Q}}{{\varvec{M}}}_{k}\right)}^\text{T}+f\left({{\varvec{a}}}^{(t)}\right)\right)\times {{\varvec{Q}}{\varvec{M}}}_{v}\end{array}$$

where ${{\varvec{T}}}^{(t)}$ represents the features related to the ROI at iteration $t$ , ${{\varvec{M}}}_{q}$ is the weight matrix employed to project features of ${{\varvec{T}}}^{(t)}$, ${\varvec{Q}}$ denotes the feature map from the encoder. ${{\varvec{M}}}_{k}$, ${{\varvec{M}}}_{v}$ represent weight matrices used to project the feature map ***Q*** into key space and value space, respectively. $f\left({{\varvec{a}}}^{(t)}\right)$ is a function that modifies the attention mechanism based on the coarse prediction mask to constrain attention only within foreground pixels, preventing irrelevant regions from influencing the refinement process.

After the last refinement iteration, the updated segmentation candidates ${{\varvec{T}}}^{(t)}$ are used to produce the final binary segmentation map ${{\varvec{a}}}^{(t)}$, ensuring well-defined object boundaries. To assign class labels to each predicted mask, we apply a linear transformation using a weight matrix ${{\varvec{M}}}_{{f}_{c}}$:

5 $${\varvec{L}}={{\varvec{T}}}^{(t )}\varvec{M}_{{f}_{c}}$$

where ${{\varvec{M}}}_{{f}_{c}} \in {\mathbb{R}}^{D\times R}$ which maps the refined features ${{\varvec{T}}}^{(t)}$ to the output class logits ${\varvec{L}} \in {\mathbb{R}}^{D \times R}$.

The final class label $\varvec{\widehat{R}}$ for each segmentation candidate is determined in Eq. (6).

6 $$\begin{array}{c}\widehat{{\varvec{R}}}=\arg\max\limits_{i=1,2,\dots,i-1} \varvec{L}\end{array}$$

where $i$ symbolizes the label index and $\widehat{{\varvec{R}}}$ mapped with the predicted masks are mathematically shown in Eq. (7).

7 $$\begin{array}{c}{{\varvec{a}}}^{(t)}= \widehat{{\varvec{R}}}\in {\mathbb{R}}^{A}\end{array}$$

**Fig. 7:**
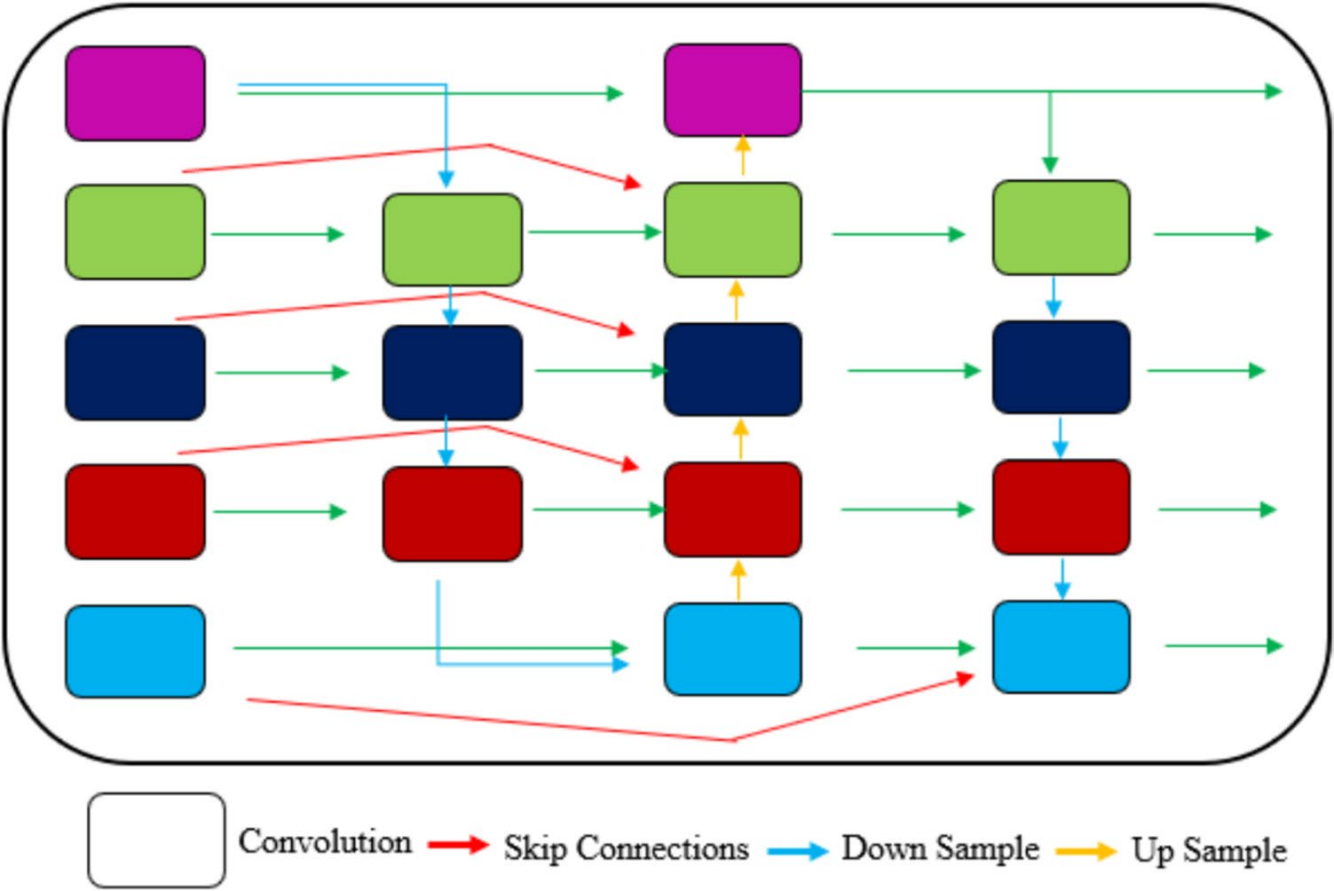
Structure of BiFPN layers

*Hierarchical Feature Integration with BiFPN*. One of the key challenges in medical image segmentation is the effective representation and processing of multi-scale features. Traditional methods often rely on pyramidal feature hierarchies extracted from backbone networks to make direct predictions. To address this, [51] introduced BiFPN, which incorporates learnable weights to determine the significance of different input features as illustrated in Fig. 7.

In this study, we integrate BiFPN with the proposed architecture to enhance multi-scale feature extraction and improve segmentation performance in medical imaging. Traditional segmentation networks such as U-Net primarily relies on skip connections to fuse high-resolution spatial features from the encoder with the semantic-rich features of the decoder. However, conventional skip connections often fail to effectively capture the significance of different features at varying scales, leading to suboptimal segmentation. Our objective is to identify a transformation function that can efficiently map features at various semantic levels and generate diverse and informative scale-invariant semantic characteristics. The thorough explanation of the BiFPN model is given below:

The set of multi-scale features, denoted by ${{\varvec{F}}}^{(i)}={{\varvec{F}}}_{1},{{\varvec{F}}}_{2}, \dots {{\varvec{F}}}_{7}$, is utilized in this method, where ${{\varvec{F}}}^{(i)}$ indicates the feature at tier $i$. The proposed model incorporates 2 to 7 tiers of input features ${{\varvec{F}}}^{(i)}={{\varvec{F}}}_{1}^{(i)},$ ${{\varvec{F}}}_{2}^{(i)}$, ${{\varvec{F}}}_{3}^{(i)},\dots ,$ where ${{\varvec{F}}}^{(i)}$ symbolizes a feature tier. For instance, if the input image size is 256 $\times$ 256, then ${{\varvec{F}}}_{3}$ corresponds to the feature layer with a resolution of $64 \times 64 \left(\frac{256}{{2}^{3}}\right)$ and ${{\varvec{F}}}_{6}^{(i)}$ denotes the input feature layer with a resolution of $8 \times 8 \left(\frac{256}{{2}^{6}}\right)$, as illustrated in Fig. 7. A weighted feature fusion approach is employed to integrate feature layers with varying resolutions, where each input is assigned a weight that can be adjusted by the network to calibrate the fusion weight of different inputs. The fusion formula is shown in Eq. (8) as follows:

8 $${\varvec{U}}=\sum_{i}\frac{{w}_{p}}{\epsilon +{\sum }_{q}{w}_{q}}.{{\varvec{I}}}_{p}$$

In order to ensure that the fusion weights are non-negative, ReLU is applied to each weight parameter ${w}_{p}$ with a small constant epsilon (ϵ = 0.0001). As depicted in Fig. 7, the feature fusion pyramid is comprised of basic units represented by blue box, which are repeatedly applied to form the entire network. As an illustration, a subsection of the network is presented for the purpose of clarity. Specifically, the first iteration is chosen as an example since the resulting $\varvec{F}^\text{o}$ does not require fusion with directly connected layers. The process of merging features of the upper and lower layers by the ${{\varvec{F}}}_{3}$ layer is illustrated as follows.

$${{\varvec{F}}}_{3}^{\text{o}}=\text{Conv}\left(\frac{{w}_{1}{{\varvec{F}}}_{3}^\text{int}+{w}_{2}{{\varvec{F}}}_{4}^\text{int}}{{w}_{1}+{w}_{2}+\epsilon }\right)$$

$${\varvec{F}}_{3}^{{\text{o}}} = {\text{Conv}}\left( {\frac{{w^{\prime}_{1} {\varvec{F}} + w^{\prime}_{2} {\varvec{F}}_{3}^\text{int} + w^{\prime}_{3} {{\varvec{F}}_{2}^\text{o} } }}{{w^{\prime}_{1} + w^{\prime}_{2} + w^{\prime}_{3} + \varepsilon }}} \right)$$

$${{\varvec{F}}}_{3}^{\text{o}}=\text{Swish}\left(\varvec{F}_{3}^{\text{o}}\right)$$

In the proposed approach, we utilize intermediate feature ${{\varvec{F}}}_{3}^\text{int}$ and output feature ${{\varvec{F}}}_{3}^\text{o}$ of the third layer. To match the resolution with the ${{\varvec{F}}}_{3}$ layer, we employ deconvolution for ${{\varvec{F}}}_{4}^\text{int}$. The output feature ${{\varvec{F}}}_{3}^\text{o}$ is activated by the Swish [17] activation function after the unification operation. For feature unification, we use the depth-wise separable convolution [18] and incorporate batch normalization after each convolution.

In order to attenuate the effects of noise, minimize computational overhead, and selectively enhance specific features, we have incorporated a feature optimization strategy. The optimization strategy is designed to attenuate the response of irrelevant regions and emphasize the features. It comprises of a 1 $\times$ 1 convolution layer, followed by normalization, a sigmoid function, and a dropout function.

##### Integrated Loss Functions

The choice of a loss function is crucial for achieving optimal performance in deep neural networks, particularly in medical imaging tasks where ROIs are significantly smaller than healthy areas; there are variations in size, and the dataset is highly imbalanced. Traditional loss functions, such as Dice loss, Binary Cross-Entropy (BCE) loss, and Hausdorff Distance (HD) loss, are widely employed in medical image segmentation; however, they often fail to effectively address severe class imbalance. Dice loss, for instance, optimizes overlaps and can mitigate class imbalance to some extent, yet it tends to underemphasize small ROIs due to its sensitivity to class size, exhibiting instability and gradient-related challenges when handling very small ROIs. Conversely, BCE assigns equal weight to all pixels, which can introduce a bias toward the majority class and exacerbate imbalance issues.

HD measures the maximum distance between boundaries of predicted and ground truth masks; even a single pixel misalignment can disproportionately affect the loss value.

Recent research has shown that combining different types of loss functions can positively affect the performance of deep learning models designed for even complex task [52].

Motivated by this, we tried to investigate multiple loss functions to determine which combination is most effective for segmenting highly complex and imbalanced MRI datasets. We proposed an integrated loss function which combines four loss functions (Dice (${D}_{\text{loss}}$), Binary (${B}_{\text{loss}}$), combined (${C}_{\text{loss}}$) and recall loss (${R}_{\text{loss}}$)) to tackle the class imbalancing in MRI images datasets, mathematically illustrated as follows.

9 $$\begin{array}{c}{I }_{\text{loss}}=\alpha {D}_{\text{loss}}+\beta {C}_{\text{loss}}+\delta {R}_{\text{loss}}+\gamma {B}_{\text{loss}}\end{array}$$

where *α*, *β*, *δ* and *γ* denote the weight for each loss function. Each term is tuned to emphasize the minority class performance. Our approach is theoretically grounded in the need to address two key limitations of conventional losses: insufficient focus on minority class recalls and poor handling of multi-scale contextual imbalance.

The Dice loss components, split for foreground (Eq. (10)) and background (Eq. (11)), ensure overlap optimization for both classes:

10 $$\begin{array}{c}{D}_{\text{F,loss}}=1-\frac{2{\sum }_{a=1}^{b}{S}_{a,0}{f}_{a,0}}{{\sum }_{a=1}^{b}{S}_{a,0}+{\sum }_{a=1}^{b}{f}_{a,0}}\end{array}$$

where $b$ denotes the total number of pixels, ${S}_{a}$ and ${f}_{a}$ correspond to the predicted mask and the ground truth mask s for the $a\text{th}$ pixel in the background class.

11 $$\begin{array}{c}{D}_{\text{B,loss}}=1-\frac{2{\sum }_{a=1}^{b}{S}_{a,1}{f}_{a,1}}{{\sum }_{a=1}^{b}{S}_{a,1}+{\sum }_{a=1}^{b}{f}_{i,1}}\end{array}$$

The combined background and foreground loss function is mathematically represented as shown in Eq. (12).

12 $$\begin{array}{c}{C}_{\text{loss}}={D}_{\text{F,loss}}+{D}_{\text{B,loss}}\end{array}$$

This dual Dice formulation captures class-specific overlap but alone may not prioritize minority class detection under severe imbalance (e.g., lesion pixels <10%). To address this issue and ensure that the model accurately detects minority classes, we have developed a recall loss function defined separately for foreground and background. This loss function emphasizes the detection of a high proportion of true positives.

13 $$\begin{array}{c}{R}_{\text{F,loss}}=1-\frac{2{\sum }_{a=1}^{b}{p}_{a,1}{S}_{a,1}}{{\sum }_{a=1}^{b}{p}_{a,1}}\end{array}$$

14 $${R}_{\text{B,loss}}=1-\frac{2{\sum }_{a=1}^{b}{S}_{s,0}{S}_{a,0}}{{\sum }_{a=1}^{b}{S}_{a,0}}$$

Here, ${p}_{a,1}$ denotes true positives for the foreground, emphasizing high recall (true positive rate) to counter the tendency of conventional losses to overlook small ROIs—a critical requirement in medical diagnostics where missing lesions are unacceptable.

The integrated loss ${R}_{\text{loss}}$ is mathematically shown as

15 $$\begin{array}{c}{R}_{\text{loss}}={R}_{\text{F,loss}}+{R}_{\text{B,loss}}\end{array}$$

We applied the loss function hyperparameters as specified in the original studies and adjusted them to optimal values. Unlike standalone traditional losses, our integrated approach leverages Dice for overlap, Recall for minority class sensitivity, Binary for stability, and Combined loss for contextual harmony, theoretically justified by their synergy in addressing multi-faceted imbalance (e.g., small ROIs in LiTS, nodules in DDTI). For a fair comparison in this study we have used three different losses, i.e., unified focal loss, combo loss and proposed integrated loss finding that the latter better balances precision and recall on highly imbalanced datasets.

### Training Details

We implemented the proposed model using the TensorFlow framework in Python 3.7, along with Matplotlib and OpenCV, on an NVIDIA RTX 4090 GPU with 24 GB of memory. We selected a batch size of 16 and used the SGD optimizer with a learning rate of 0.01, momentum of 0.9, and weight decay of 0.0001. To minimize the impact of overfitting and improve the model's generalization ability and robustness, we employed data augmentation techniques as explained in Sect. 3.2. The dataset was divided into three parts: 80% for training, 10% for testing, and 10% for validation. An early stopping mechanism was adopted to address overfitting. The model was trained for a total of 440 epochs.

## Result Analysis and Discussion

This section investigates the comparative performance of various deep learning models on MRI datasets characterized by significant class imbalances. We analyzed nine widely recognized models, frequently used in medical image segmentation, along with our newly developed 2D-UNet, to ensure a comprehensive evaluation. The performance of each model was quantitatively assessed using metrics such as IoU, Dice Similarity Coefficient (DSC), Precision, and Recall. The statistical results of these metrics are shown in Tables 2, 3, and 4. The results of each metric are given below.

**Table 2:** The performance of deep learning models on MRI datasets before integrating class-balancing mechanisms

Model	IoU	DSC	Precision	Recall	Dataset
UNet	0.875 ± 0.032	0.841 ± 0.025	0.769 ± 0.048	0.901 ± 0.052	DDTI
RCNN	0.869 ± 0.074	0.836 ± 0.029	0.759 ± 0.049	0.872 ± 0.019	
DeepLab-V3	0.924 ± 0.027	0.931 ± 0.032	0.911 ± 0.030	0.929 ± 0.047	
UNet++	0.861 ± 0.061	0.908 ± 0.052	0.899 ± 0.042	0.919 ± 0.035	
AG-Net [53]	0.881 ± 0.044	0.902 ± 0.032	0.911 ± 0.024	0.916 ± 0.040	
DANet [54]	0.831 ± 0.033	0.822 ± 0.039	0.754 ± 0.054	0.891 ± 0.060	
CE-Net [55]	0.925 ± 0.021	0.929 ± 0.041	0.920 ± 0.052	0.929 ± 0.047	
cGAN [56]	0.851 ± 0.047	0.900 ± 0.036	0.892 ± 0.042	0.902 ± 0.052	
PSPNet [57]	0.917 ± 0.029	0.911 ± 0.060	0.901 ± 0.032	0.916 ± 0.036	
Proposed model	0.935 ± 0.024	0.948 ± 0.026	0.941 ± 0.031	0.942 ± 0.031	
UNet	0.801 ± 0.059	0.795 ± 0.033	0.804 ± 0.035	0.839 ± 0.056	LiTS MICCAI 2017
RCNN	0.751 ± 0.023	0.778 ± 0.019	0.794 ± 0.048	0.832 ± 0.034	
DeepLab-V3	0.915 ± 0.044	0.928 ± 0.082	0.887 ± 0.048	0.899 ± 0.075	
UNet++	0.884 ± 0.058	0.847 ± 0.085	0.829 ± 0.052	0.880 ± 0.041	
AG-Net [53]	0.821 ± 0.013	0.815 ± 0.026	0.817 ± 0.046	0.772 ± 0.076	
DANet [54]	0.845 ± 0.042	0.831 ± 0.033	0.835 ± 0.021	0.784 ± 0.033	
CE-Net [55]	0.900 ± 0.011	0.912 ± 0.042	0.891 ± 0.037	0.892 ± 0.025	
cGAN [56]	0.855 ± 0.048	0.837 ± 0.065	0.814 ± 0.054	0.874 ± 0.034	
PSPNet [57]	0.896 ± 0.033	0.905 ± 0.059	0.885 ± 0.021	0.885 ± 0.032	
Proposed model	0.928 ± 0.040	0.936 ± 0.034	0.916 ± 0.042	0.922 ± 0.046	
UNet	0.785 ± 0.072	0.699 ± 0.045	0.701 ± 0.049	0.785 ± 0.079	BUSI
RCNN	0.698 ± 0.058	0.698 ± 0.058	0.710 ± 0.085	0.747 ± 0.043	
DeepLab-V3	0.798 ± 0.048	0.725 ± 0.040	0.805 ± 0.045	0.895 ± 0.029	
UNet++	0.749 ± 0.051	0.689 ± 0.075	0.711 ± 0.055	0.852 ± 0.047	
AG-Net [53]	0.793 ± 0.057	0.703 ± 0.033	0.711 ± 0.038	0.799 ± 0.061	
DANet [54]	0.813 ± 0.042	0.744 ± 0.041	0.773 ± 0.031	0.835 ± 0.037	
CE-Net [55]	0.825 ± 0.019	0.761 ± 0.043	0.837 ± 0.036	0.862 ± 0.054	
cGAN [56]	0.755 ± 0.029	0.743 ± 0.043	0.771 ± 0.043	0.862 ± 0.054	
PSPNet [57]	0.820 ± 0.032	0.774 ± 0.021	0.843 ± 0.035	0.865 ± 0.050	
Proposed model	0.859 ± 0.040	0.817 ± 0.038	0.891 ± 0.042	0.905 ± 0.023

**Table 3:** The comparison of various attention mechanisms for deep learning models

Model	IoU	DSC	Precision	Recall	Dataset
UNet+soft attention	0.882 ± 0.045	0.852 ± 0.026	0.775 ± 0.031	0.916 ± 0.045	DDTI
UNet+spatial	0.893 ± 0.036	0.868 ± 0.039	0.776 ± 0.039	0.931 ± 0.027	
UNet+EAM	0.928 ± 0.018	0.877 ± 0.044	0.784 ± 0.032	0.942 ± 0.031	
RCNN+soft attention	0.876 ± 0.041	0.849 ± 0.054	0.768 ± 0.043	0.910 ± 0.035	
RCNN+spatial	0.918 ± 0.041	0.860 ± 0.025	0.771 ± 0.024	0.919 ± 0.020	
RCNN+EAM	0.925 ± 0.023	0.871 ± 0.046	0.781 ± 0.033	0.929 ± 0.031	
PSPNet+soft attention	0.919 ± 0.019	0.925 ± 0.025	0.911 ± 0.017	0.926 ± 0.040	
PSPNet+spatial	0.922 ± 0.023	0.931 ± 0.032	0.924 ± 0.028	0.943 ± 0.035	
PSPNet+EAM	0.930 ± 0.027	0.942 ± 0.038	0.932 ± 0.046	0.952 ± 0.042	
RCNN+soft attention	0.769 ± 0.028	0.785 ± 0.046	0.813 ± 0.036	0.841 ± 0.042	LiTS MICCAI 2017 Dataset
RCNN+spatial	0.798 ± 0.033	0.829 ± 0.045	0.821 ± 0.019	0.852 ± 0.052	
RCNN+EAM	0.827 ± 0.029	0.834 ± 0.028	0.833 ± 0.024	0.865 ± 0.022	
UNet+soft attention	0.826 ± 0.041	0.804 ± 0.032	0.815 ± 0.039	0.844 ± 0.028	
UNet+spatial	0.833 ± 0.014	0.833 ± 0.026	0.832 ± 0.043	0.852 ± 0.049	
UNet+EAM	0.844 ± 0.046	0.839 ± 0.038	0.844 ± 0.049	0.869 ± 0.035	
PSPNet+soft attention	0.902 ± 0.017	0.919 ± 0.045	0.891 ± 0.034	0.898 ± 0.042	
PSPNet+spatial	0.925 ± 0.044	0.922 ± 0.023	0.902 ± 0.037	0.913 ± 0.028	
PSPNet+EAM	0.934 ± 0.026	0.934± 0.020	0.920 ± 0.067	0.924 ± 0.038

**Table 4:** The impact of different modules on the performance of DeepLab-V3 and UNet

Model	IoU	DSC	Precision	Recall	
DeepLab-V3+EAM+PIL	0.931± 0.019	0.935 ± 0.022	0.922 ± 0.031	0.930 ±0.041	DDTI
DeepLab-V3+EAM+PIL+Dual Decoders	0.937 ± 0.022	0.938 ± 0.031	0.933 ± 0.037	0.940 ± 0.043	
UNet+BiFPN	0.909 ± 0.034	0.914 ± 0.022	0.915 ± 0.032	0.927 ± 0.047	
UNet+BiFPN+EAM	0.917 ± 0.021	0.926 ± 0.038	0.924 ± 0.038	0.922 ± 0.032	
UNet+BiFPN+EAM+PIL	0.921 ± 0.036	0.938 ± 0.043	0.929 ± 0.032	0.930 ± 0.044	
UNet+BiFPN+EAM+PIL+Dual Decoders (2D-UNet)	0.935 ± 0.024	0.948 ± 0.036	0.941 ± 0.031	0.942 ± 0.031	
DeepLab-V3	0.915 ± 0.044	0.928 ± 0.082	0.887 ± 0.048	0.899 ± 0.075	LiTS MICCAI 2017
DeepLab-V3+EAM+PIL	0.920 ± 0.025	0.929 ± 0.034	0.892 ± 0.043	0.908 ± 0.042	
DeepLab-V3+EAM+PIL+Dual Decoders	0.921 ± 0.028	0.932 ± 0.058	0.901 ± 0.036	0.904 ± 0.045	
UNet+BiFPN	0.844 ± 0.054	0.836 ± 0.038	0.845 ± 0.028	0.850 ± 0.041	
UNet+BiFPN+EAM	0.884 ± 0.037	0.853 ± 0.048	0.869 ± 0.029	0.869 ± 0.025	
UNet+BiFPN+EAM+PIL	0.917 ± 0.021	0.906 ± 0.036	0.898 ± 0.044	0.877 ± 0.042	
UNet+BiFPN+EAM+PIL+Dual Decoders (2D-UNet)	0.923 ± 0.035	0.918 ± 0.032	0.905 ± 0.025	0.898 ± 0.028	

### Evaluation Metrics

#### Precision

Precision in the context of image segmentation refers to the ratio of correctly predicted positive observations to the total predicted positives. This metric is crucial for evaluating the performance of segmentation models. High precision indicates a lower rate of false positives. The formula for precision is given as the following:

$$\begin{array}{c}\text{Precision}=\frac{{T}^{\text{Positive}}}{{T}^{\text{Positive}}+{F}^{\text{Positive}}}\end{array}$$

Table 2 presents the performance of the models on three datasets (DDTI, LiTS MICCAI 2017, and BUSI). As in the DDTI dataset, lesion regions are underrepresented compared to the background, leading to a modest class imbalance. This imbalance affects the precision of baseline models. For example, UNet and RCNN show subdued precision scores 0.769 ± 0.048 and 0.759 ± 0.049, respectively), indicating a tendency to misclassify background areas as lesions. In contrast, the proposed model achieves the highest precision 0.941 ± 0.031, demonstrating its ability to reduce false positives by improving the distinction between lesion and non-lesion regions.

The LiTS MICCAI 2017 dataset possesses a more pronounced class imbalance, wherein tumor regions occupy a small portion of the overall image. This imbalance is conspicuously reflected in the diminished precision of UNet 0.804 ± 0.035 and RCNN 0.794 ± 0.048, both of which exhibit a proclivity to over-predict background regions and under-segment tumor areas. Although models like DeepLab-V3, UNet++, and CE-Net show improved performance, the proposed model achieves a higher precision of 0.916 ± 0.042, highlighting its ability to maintain accurate segmentation despite class imbalance.

The BUSI dataset betokens significant class imbalance, which detrimentally impacts the segmentation ability of deep learning models. For example, UNet and UNet++ attain precision scores of 0.701 ± 0.049 and 0.711 ± 0.055, respectively, evidencing their difficulties in precisely segmenting underrepresented class regions.

Likewise, DANet and cGAN showcase low precision, with 0.773 ± 0.031 and 0.771 ± 0.043 values, respectively.

We have compared the segmentation performance of models such as PSPNet and GAN-based segmentation models, including cGAN. Their precision metrics, as reported in the table, demonstrate significantly improved performance.

The suggested model, however, achieves a higher precision of 0.891 ± 0.042. This is ascribed to the multifaceted strategy that successfully addresses the problems brought on by class imbalance by highlighting underrepresented areas and enhancing the precision of lesion boundaries.

##### Recall

Recall, also known as sensitivity, measures the ability of a model to identify all true positives correctly. A high recall rate ensures that the model tests all conditions and reduces the risk of false negatives. Recall can be calculated by the following equation:

$$\begin{array}{c}\text{Recall}=\frac{{T}^{\text{Positive}}}{{T}^{\text{Positive}}+{F}^{\text{Negative}}}\end{array}$$

For the DDTI dataset, the RCNN model achieves a recall of 0.872 ± 0.019, indicating moderate sensitivity. UNet and DeepLab-V3 exhibit improved recall values of 0.901 ± 0.052 and 0.929 ± 0.047, respectively, indicating their greater ability to recognize true positives. UNet++ further boosts sensitivity with a recall of 0.919 ± 0.035, while AG-Net and CE-Net achieve competitive recalls of 0.916 ± 0.040 and 0.929 ± 0.047, respectively. The cGAN model also performs well, with a recall of 0.902 ± 0.052. PSPNet records a recall of 0.916 ± 0.036, demonstrating its robust capability in pinpointing lesion areas. Nevertheless, the proposed model surpasses all contenders, securing the top recall of 0.942 ± 0.031, which emphasizes its exceptional strength in reducing missed detections and thoroughly mapping lesion regions.

In the LiTS MICCAI 2017 dataset, UNet and RCNN achieves a recall of 0.839 ± 0.056 and 0.832 ± 0.034, respectively. AG-Net and UNet++ eke out slight gains, attaining recall scores of 0.772 ± 0.076 and 0.880 ± 0.040. DeepLab-V3 provides a more consistent and higher recall of 0.899 ± 0.075, while CE-Net and cGAN achieve recalls of 0.892 ± 0.025 and 0.874 ± 0.034, respectively. PSPNet obtains highly accurate results with a recall of 0.885 ± 0.032. Once more, the proposed model rises above the fray, clinching a recall of 0.922 ± 0.046, a testament to its resilience in identifying lesions even in imbalanced datasets.

For the BUSI dataset, UNet and RCNN show subdued recall values of 0.785 ± 0.079 and 0.747 ± 0.043, compared to other segmentation models. UNet++ and AG-Net moderate recalls of 0.852 ± 0.047 and 0.799 ± 0.061, respectively. CE-Net and PSPNet demonstrate greater sensitivity with recalls of 0.862 ± 0.054 and 0.865 ± 0.050, respectively, while DANet and cGAN attain recalls of 0.835 ± 0.037 and 0.862 ± 0.054, respectively. DeepLab-V3's recall of 0.895 ± 0.029 represents a notable improvement. Even in difficult class imbalance scenarios, the proposed model consistently identifies lesion regions in breast ultrasound images, achieving the highest recall of 0.905 ± 0.023.

##### IoU

IoU quantifies the overlap between the predicted segmentation and the ground truth, providing a measure of the accuracy of the model. Specifically, IoU is the ratio of the intersection of the predicted and actual positive regions to the union of these regions. A higher IoU indicates a more accurate segmentation. The mathematical formula of IoU is given as follows:

$$\begin{array}{c}\text{IoU}=\frac{{2T}^{\text{Positive}}}{{2T}^{\text{Positive}}+ {F}^{\text{Positive}}+{F}^{\text{Negative}}}\end{array}$$

UNet and DeepLab-V3 show better performance with IoU values of 0.801 ± 0.059 and 0.924 ± 0.027, respectively, demonstrating their efficacy in accurate thyroid image segmentation, while RCNN achieves a noteworthy IoU of 0.869 ± 0.074 for the DDTI Dataset. With a formidable IoU of 0.935 ± 0.024, 2D-Unet transcends all others, whilst UNet++ performs marginally worse with an IoU of 0.861 ± 0.061. CE-Net also performs well, outperforming DANet (0.831 ± 0.033) and AG-Net (0.881 ± 0.044) with an IoU of 0.925 ± 0.021. With IoU values of 0.917 ± 0.029 and 0.851 ± 0.047, respectively, PSPNet and cGAN also demonstrate competitive results, highlighting the resilience of sophisticated architectures in thyroid image segmentation.

In the LiTS MICCAI 2017 Dataset, RCNN and UNet++ show some improvement but there is still an opportunity for improvement in segmentation accuracy, with IoU values of 0.751 ± 0.023 and 0.884 ± 0.058, respectively. DeepLab-V3 performs strongly with an IoU of 0.915 ± 0.044, while the proposed 2D-Unet achieves an even higher IoU of 0.928 ± 0.040, demonstrating its superior segmentation capability. CE-Net also excels with an IoU of 0.900 ± 0.11, outperforming DANet (0.845 ± 0.042) and AG-Net (0.821 ± 0.013). PSPNet and cGAN demonstrate competitive performance, confirming the models' efficacy in liver tumor segmentation.

In the case of BUSI, extensively used segmentation models such as UNet and UNet++ stumble with IoU scores of 0.785 ± 0.072 and 0.749 ± 0.051, respectively, indicating problems with precisely segmenting breast lesions.

The need for better segmentation models in breast imaging is further highlighted by RCNN's modest IoU of 0.698 ± 0.058. With a higher IoU of 0.825 ± 0.019, CE-Net outperforms UNet, UNet++, and RCNN, showcasing its superior target region segmentation capabilities. CE-Net outperforms DeepLab-V3 (0.798 ± 0.048) and AG-Net (0.793 ± 0.057), while DANet performs competitively with an IoU of 0.813 ± 0.042. Yet, the suggested model's improved segmentation ability is demonstrated by its highest IoU of 0.859 ± 0.040. PSPNet and cGAN also deliver respectable performances, with IoU values of 0.820 ± 0.032 and 0.755 ± 0.029, respectively, further emphasizing the potential of advanced architectures in addressing the challenges of breast ultrasound image segmentation.

##### DSC

DSC is a statistical tool used to measure the similarity between generated images and ground truth. In the context of image segmentation, DSC measures the overlap between the predicted segmentation and the ground truth. It is calculated as the ratio of twice the area of overlap between the prediction and the ground truth to the sum of the areas of the prediction and the ground truth. The formula for DSC is given as

$$\begin{array}{c}\text{DSC}=\frac{{T}^{\text{Positive}}}{{T}^{\text{Positive}}+ {F}^{\text{Positive}}+{F}^{\text{Negative}}}\end{array}$$

For the DDTI Dataset, UNet obtains a DSC of 0.841 ± 0.025, whereas RCNN achieves a DSC of 0.836 ± 0.029. DeepLab-V3, with a DSC of 0.931 ± 0.032, exhibits superior precision in capturing intricate pathological features, outperforming many contemporary models. UNet++ steps up too, hitting a DSC of 0.908 ± 0.052, and CE-Net reaches a DSC of 0.929 ± 0.041, prevailing over DANet and AG-Net (0.902 ± 0.032). DANet and DeepLab-V3 exhibit competitive results, with DSCs of 0.822 ± 0.039 and 0.931 ± 0.032, respectively. However, the proposed model tops them all with a DSC of 0.948 ± 0.026, making it the best at tackling segmentation challenges.

On the LiTS MICCAI 2017 dataset, RCNN achieves a DSC of 0.778 ± 0.019, reflecting its reasonable performance in liver tumor segmentation. UNet and UNet++ exhibit reasonable improvements, with DSCs of 0.795 ± 0.033 and 0.847 ± 0.085, respectively. However, their performance decreases on BUSI, with DSCs of 0.689 ± 0.075 and 0.698 ± 0.058, respectively, indicating constraints in generalizing across datasets. AG-Net and DANet achieves DSCs of 0.815 ± 0.026 and 0.831 ± 0.033, respectively. PSPNet rolls out a strong DSC of 0.905 ± 0.059, whereas cGAN notches 0.837 ± 0.065, and CE-Net records 0.912 ± 0.042. 2D-UNet tops the pack, snagging the best DSC of 0.936 ± 0.034 on this dataset.

For BUSI, RCNN and DeepLab-V3 achieves a DSC of 0.699 ± 0.045 and 0.805 ± 0.045, respectively. DSCs of 0.711 ± 0.055 and 0.711 ± 0.038 are obtained by AG-Net and UNet++, respectively. DANet and CE-Net outperform with DSCs of 0.773 ± 0.031 and 0.837 ± 0.036, respectively. The cGAN's DSC of 0.771 ± 0.043 reflects its limitations, and PSPNet's DSC of 0.843 ± 0.035 demonstrates its competitive advantage. 2D-UNet demonstrates the capacity to generalize across datasets and retain excellent segmentation.

## Discussion

Initially, we conducted experiments without implementing any class-balancing mechanisms to establish a baseline for comparison. This analysis included three datasets, i.e., DDTI, LiTS MICCAI 2017, and BUSI. The complete statistical results are shown in Table 2. Among the traditional models, UNet++ demonstrates a better ability for feature extraction. Throughout our evaluation, the five-fold cross-validation method was employed to ensure the reliability and reproducibility of the results. This detailed examination underlines the differential capabilities of each model, highlighting that while UNet provides a strong baseline, models like DeepLab-V3 and UNet++ offer significant improvements in handling complex medical imaging tasks across multiple datasets.

### Impact of EAM

Table 3 presents a comparison of the performance of extant deep learning-based image segmentation models across two datasets: DDTI and LiTS MICCAI 2017. Table 3 illustrates the performance scores following the integration of three attention mechanisms, namely soft attention, spatial attention, and EAM, into UNet, RCNN, and PSPNet. The results consistently demonstrate that the use of attention mechanisms amplifies the model’s segmentation ability across all evaluation metrics on both datasets. EAM offers the highest performance gains, indicating its preeminent ability to discern intricate spatial dependencies and multi-scale contextual features. On the DDTI dataset, UNet augmented with soft attention attains an IoU of 0.882 ± 0.045, DSC of 0.852 ± 0.026, Precision of 0.775 ± 0.031, and Recall of 0.916 ± 0.045. Similarly, RCNN with soft attention obtains IoU of 0.876 ± 0.041, DSC of 0.849 ± 0.054, Precision of 0.768 ± 0.043, and Recall of 0.910 ± 0.035. The introduction of spatial attention further improves performance, as demonstrated by RCNN with spatial attention, which achieves an IoU of 0.918 ± 0.041 and a DSC of 0.860 ± 0.025, indicating that spatial attention assists in refining spatial localization and boundary delineation. The most notable performance gains are observed with the integration of EAM. For instance, UNet with EAM reaches the pinnacle of performance with IoU of 0.928 ± 0.018, DSC of 0.877 ± 0.044, Precision of 0.784 ± 0.032, and Recall of 0.942 ± 0.031, reflecting EAM's superior capability to emphasize salient regions while minimizing background noise. Among all models, PSPNet with EAM demonstrates the best overall score on the DDTI dataset, achieving IoU of 0.930 ± 0.027, DSC of 0.942 ± 0.038, Precision of 0.932 ± 0.046, and Recall of 0.952 ± 0.042. This improvement can be attributed to PSPNet's capacity to extract multi-scale contextual information, which, when combined with EAM, enhances both local and global feature representations.

In the comparatively challenging LiTS MICCAI 2017 dataset, the efficacy of attention mechanisms persists, albeit with fluctuating outcomes across different models. RCNN, equipped with soft attention, attains IoU of 0.769 ± 0.028, whereas UNet with soft attention demonstrates an improved IoU of 0.826 ± 0.041.

The incorporation of spatial attention confers additional refinement, with RCNN augmented by spatial attention attaining an IoU of 0.798 ± 0.033 and a DSC of 0.829 ± 0.045. Yet again, EAM yields the most pronounced advancements across all evaluative indices. For instance, UNet with EAM achieves IoU of 0.844 ± 0.046 and DSC of 0.839 ± 0.038, whereas RCNN+EAM secures IoU of 0.827 ± 0.029 and DSC of 0.834 ± 0.028. PSPNet+EAM exhibits the pinnacle of segmentation precision on the LiTS MICCAI 2017 dataset, registering IoU of 0.934 ± 0.026, DSC of 0.934 ± 0.020, Precision of 0.920 ± 0.067, and Recall of 0.924 ± 0.038.

### Impact of PIL

PIL is crucial in refining the segmentation process by utilizing semantic and spatial information to accurately guide up-sampled high-level feature maps. For the DDTI dataset, integrating PIL into the DeepLab-V3+EAM model results in an increase in IoU from 0.924 ± 0.027 to 0.931 ± 0.019 and a rise in DSC from 0.931 ± 0.032 to 0.935 ± 0.022. Similarly, the precision improves from 0.911 ± 0.030 to 0.922 ± 0.031 and recall from 0.929 ± 0.047 to 0.930 ± 0.041. When PIL is combined with EAM and Dual Decoders in DeepLab-v3, we observe further enhancements, with IoU climbing to 0.937 ± 0.022, DSC to 0.938 ± 0.031, Precision to 0.933 ± 0.037, and Recall to 0.940 ± 0.043. In the case of the LiTS MICCAI 2017 dataset, the impact of PIL remains better. DeepLab-V3+EAM+PIL performance is good, with IoU increasing to 0.896 ± 0.284 and DSC to 0.862 ± 0.587. The UNet model, with Modified Skip Connections, EAM, PIL, and Dual Decoders, shows an IoU of 0.923 ± 0.485 and a DSC of 0.918 ± 0.322. These results show PIL's efficiency in enhancing model performance by providing a more robust and precise integration.

### Impact of Dual Decoder

For the DeepLab-V3 model, integrating the dual decoder architecture enhances the segmentation scores on the DDTI dataset. DSC improves from 0.908 ± 0.043 to 0.938 ± 0.031, IoU increases from 0.892 ± 0.017 to 0.903 ± 0.021, Precision increases from 0.941 ± 0.019 to 0.956 ± 0.042, and Recall from 0.952 ± 0.023 to 0.963 ± 0.035. These improvements highlight the dual decoder's capability to enhance feature capture significantly.

Further, when assessing the UNet model, particularly with BiFPN, EAM, and PIL enhancements, the addition of dual decoders (2D-UNet) provides considerable performance across both DDTI and LiTS MICCAI datasets. Specifically, in the DDTI dataset, the dual decoder UNet achieves IoU improvement to 0.935 ± 0.024, DSC to 0.948 ± 0.036, Precision to 0.941 ± 0.031, and Recall to 0.942 ± 0.031. In the LiTS MICCAI 2017 dataset, 2D-UNet demonstrates IoU of 0.923 ± 0.035, DSC of 0.918 ± 0.322, Precision of 0.905 ± 0.475, and Recall of 0.898 ± 0.258.

### Impact of Different Loss Functions

Table 5 delineates the comparative evaluation of different loss functions across the LiTS MICCAI 2017 and DDTI datasets, showing their impact on segmentation performance. Particularly, the proposed hybrid loss function significantly enhances the performance metrics, demonstrating the effectiveness of loss functions in complex segmentation tasks. For the LiTS MICCAI 2017 dataset, the proposed hybrid loss function achieves IoU of 0.847 ± 0.047, DSC of 0.958 ± 0.051, Precision of 0.964 ± 0.031, and Recall of 0.969 ± 0.082. In contrast, within the DDTI dataset, the proposed hybrid loss function again demonstrates strong performance with DSC of 0.925 ± 0.682 and a high Recall of 0.934 ± 0.526. The combo loss shows a slightly higher IoU of 0.861 ± 0.254 and comparable DSC and precision metrics. This suggests that while the proposed hybrid loss excels in certain aspects, the combo loss might be more effective in specific contexts within this dataset due to the different characteristics and challenges presented by DDTI.

**Table 5:** The impact of loss functions on the segmentation performance of 2D-UNet

Loss functions	IoU	DSC	Precision	Recall	Dataset
Proposed hybrid loss	0.847 ± 0.047	0.958 ± 0.051	0.964 ± 0.031	0.969 ± 0.082	LiTS MICCAI 2017
Unified focal loss	0.829 ± 0.047	0.947 ± 0.023	0.954 ± 0.041	0.948 ± 0.042	
Combo loss	0.874 ± 0.035	0.963 ± 0.044	0.954 ± 0.043	0.954 ± 0.021	
Proposed hybrid loss	0.812 ± 0.852	0.925 ± 0.682	0.914 ± 0.682	0.934 ± 0.526	DDTI
Unified focal loss	0.847 ± 0.759	0.899 ± 0.523	0.902 ± 0.654	0.914 ± 0.586	
Combo loss	0.861 ± 0.254	0.905 ± 0.485	0.896 ± 0.632	0.935 ± 0.584	

## Conclusion

Class imbalance presents a significant challenge in the training and performance of deep learning models, particularly within the domain of medical image segmentation using highly imbalanced datasets. In this study, we have developed a comprehensive strategy to address these challenges, focusing on improving segmentation accuracy through innovative data augmentation, algorithmic adjustments, and architectural innovations. We adapted the RandAugment method specifically for medical imaging data to enhance the diversity and quality of our training sets. We have introduced novel attention mechanisms such as EAM, soft attention and spatial attention, which help the model focus on specific features while ignoring irrelevant ones. Additionally, the integration of BiFPN and dual decoder architecture allows the model to capture and process distinct aspects of input representations, i.e., foreground and background, simultaneously. This is further improved by the integration of PIL, which enhances the fusion of high-level and low-level features. The collective impact of these techniques has been validated across multiple datasets using IoU, Dice Similarity Coefficient, Precision, and Recall. These enhancements are prominent in datasets such as DDTI and LiTS MICCAI 2017, which are characterized by their complexity and the high degree of class imbalance.

## Data Availability

The data used in this study is publicly available.
